# Preliminary study on designing the binder of sperm-1 synthetic vaccine using sequence-based methods and molecular docking

**DOI:** 10.5455/javar.2022.i576

**Published:** 2022-03-10

**Authors:** Wayan Wariata, Made Sriasih, Anwar Rosyidi, Muhamad Ali, Sulaiman Ngongu Depamede

**Affiliations:** Faculty of Animal Science, University of Mataram, Mataram, Indonesia

**Keywords:** BSP1, epitope, peptide, ruminant, synthetic vaccine, T-cell

## Abstract

**Objective:**

The main objective of this study is to design a synthetic vaccine from the binder of sperm-1 (BSP1).

**Materials and Methods:**

This study was carried out using bioinformatics-related techniques. BSP-1 has been chosen as one of the biomarkers of a ruminant’s male fertility. We hypothesize that the BSP1 synthetic vaccines, which contain T-cell epitopes, can produce antibodies more effectively for the development of a sperm fertility detection kit. A sequence of BSP-1 peptides A0A0K1YXR5 from *Bubalus bubalis* (Domestic water buffalo) origin has been decided to be used to develop the peptide vaccine.

**Results:**

In this study, we succeeded in making synthetic vaccines from BSP-1 with a peptide sequence of LPEDSVPDEERVFPFTYRNRKHF. The three-dimensional theoretical prediction analysis of the peptide binding pattern to its ligand, as well as the molecular docking, has also been revealed.

**Conclusions:**

A synthetic vaccine from the BSP-1 has been developed in this study with the amino acid sequence LPEDSVPDEERVFPFTYRNRKHF, which is buffer-soluble, and the three-dimensional theoretical prediction analysis of the peptide binding pattern of BSP-1 to its ligand, as well as molecular docking, has also been revealed.

## Introduction

In a sustainable cattle industry, bull fertility plays a central role. Subfertile bulls might result in serious economic losses. As a result, efforts to develop a bull fertility detection device are continuously being carried out, including the development of peptide biomarker-based immunoassay methods. Since the early 2020s, the utilization of synthetic peptide antigens/immunogens to produce specific immunological reagents for specific purposes, including for studying animal fertility, has increased significantly [1–4]. One of the processes in designing synthetic peptides involves bioinformatics [3,5]. The purpose of this study was to determine how to utilize bioinformatics-related techniques to design peptide vaccines. In this study, we used the binder of sperm-1 (BSP-1) peptide as a model. BSP-1 is a family of proteins found in several species‘ seminal plasma (SP) [6]. BSP-1 can stabilize the sperm membrane and mediate the sperm‘s binding to the oviduct‘s epithelium [7,8]. Therefore, BSP-1 has been chosen as one of the biomarkers of male fertility. The research in an effort to find biomarkers for male fertility selection both proteomically and genomically has been carried out systematically for the last two decades [9,10]. These can be based on molecular compounds in sperm and SP [10–13]. Many promising biomarkers for bull fertility have been studied based on the proteomic landscape of SP [14,15]. A recent publication using matrix-assisted laser desorption ionization-time of flight/mass spectrometry (MALDI-TOF/MS) technology has identified 23 proteins associated with the fertility levels expressed in the spermatozoa of crossed bulls [16]. According to them, of the 23 protein biomarkers, there are two biomarkers with the most potential, namely the peptide Enolase-1 as a marker that a male is fertile, and BSP-1 as a marker that a male is infertile or sterile [16].

The MALDI-TOF/MS method is good for proteomic studies, including a study on bulls’ fertility or infertility, as has been done by Aslam et al. [16]. However, this method is relatively expensive and quite complicated. One of the ways that is quite straightforward is to use a peptide sequence that can be accessed by applying bioinformatics techniques. Then the peptide sequence obtained will be further analyzed to be used to make a vaccine. The antibodies obtained can then be used to explore the expression of these peptides immunologically [3]. Success in producing antibodies against synthetic peptide antigens is currently very important and needed not only for research purposes but also for the biotech industry‘s purposes. Several essential actions must be taken, including the T-cell epitope prediction of the target protein or peptide, in this study, the BSP-1. For this reason, a study using sequence-based and molecular docking methods was conducted.

## Materials and Methods

This study was mainly software-based experimental research; hence, the approval of the relevant ethics committee was not necessarily implied. The main material of this research is BSP-1, with amino acid sequences derived from tracking on protein banks such as ExPASy and UniProtKB (https://www.uniprot.org/), according to Depamede [17]. The retrieved sequences were aligned using Clustal Omega (https://www.ebi.ac.uk/Tools/msa/clustalo/; [18]. Prediction of the availability of B and T cell epitopes was conducted using the Immune Epitope Database (IEDB) (http://www.iedb.org/) and, when available, the prospective B and T cell epitopes were determined based on the conserved epitopes found [19]. In addition, to find peptides binding to major histocompatibility complex (MHC) class I and class II molecules, two servers, i.e., ProPred-I and ProPred-Server, were adopted [20,21]. In addition, the peptide sequences were also evaluated using NetMHCpan-4.1 (http://www.cbs.dtu.dk/services/NetMHCpan/index_4.1a.php), [22,23]. Finally, the theoretical prediction of the binding of BSP-1 with its ligands was carried out based on the I-TASSER software program [24].

## Results

**Figure 1.**
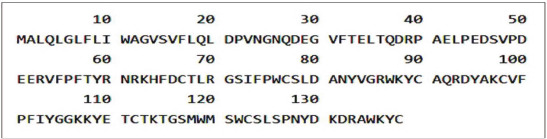
Amino acid sequence of BSP1 belongs to *B. bubalis* (Domestic water buffalo) retrieved from UniProtKB (https://www.uniprot.org/).

**Figure 2.**
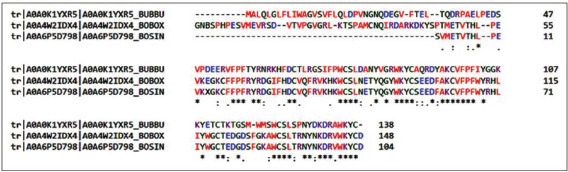
Multiple sequence alignment of Domestic water buffalo BSP1 (A0A0K1YXR5) against cattle binder sperm protein using Clustal Omega. Conserved residues are denoted by star (*).

By typing BSP1, the tracking results on the Protein Bank data, namely, on ExPASy and UniProtKB, were found to have 170 entry identifier numbers. Furthermore, when retrieved based on species or organism, it found 11 entries (Table 1). BSP1 with entry identifier A0A0K1YXR5is belonging to *Bubalus bubalis* (Domestic water buffalo) consists of 38 residues (Fig. 1). We then conducted multiple alignments of the A0A0K1YXR5 hybrid cattle (*Bos indicus* × *Bos taurus*) with gene name binder of sperm protein homolog 1 (BSPH1), entry identifier A0A4W2IDX4, and *B. indicus* (Zebu) A0A6P5D798, as the cattle (*Bos*) representative, and the result is presented in Figure 2.

**Figure 3.**
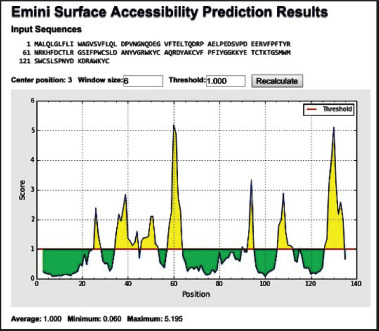
The results of the analysis of the BSP-1 Epitope prediction based on the IEDB. Predicted Emini surface accessibility of the proposed epitope, with a minimum propensity score of 0.060 and a maximum score of 5.195. Sequence positions and surface probabilities are directed by the *X*- and *Y*- axes, respectively. The areas above the threshold are antigenic, shown in yellow.

This study aimed to design a peptide vaccine to produce BSP1 antibodies. Work on finding epitopes associated with vaccine design is increasingly dependent on bioinformatics analytical tools and access to data stores pertinent to pathogens and specific immune reactions [24,25]. This requires a combination of techniques or methods. Based on these considerations, in this study, the IEDB program was also used based on Droppa-Almeida et al. [26]. This is shown in in Figure 3.

**Table 1.** Binder sperm protein in ruminants and its entry identifier retrieved from UnibProtKB.

No.	Entry identifier	UniProtKB entry identifier	Protein names	Gene name	Organism	Sequence number
1	A0A6P3I827	A0A6P3I827_BISBI	BSPH1	BSPH1	*Bison bison bison*	115
2	A0A4W2IDX4	A0A4W2IDX4_BOBOX	BSPH1	BSPH1	*B. indicus* × *B. tauru*s (Hybrid cattle)	138
3	A0A4W2CI70	A0A4W2CI70_BOBOX	BSPH1		*B. indicus* × *B. taurus* (Hybrid cattle)	132
4	A0A4W2D2A3	A0A4W2D2A3_BOBOX	BSPH1		*B. indicus* × *B. taurus* (Hybrid cattle)	138
5	A0A4W2GYS9	A0A4W2GYS9_BOBOX	BSPH1	BSPH1	*B. indicus* × *B. taurus* (Hybrid cattle)	132
6	B3EWK6	SFP1_BOSIN	SP protein PDC-109		*B. indicus* (Zebu)	32
7	A0A6P5D798	A0A6P5D798_BOSIN	BSPH1	BSPH1	*B. indicus* (Zebu)	102
8	A0A0M4B6N2	A0A0M4B6N2_BUBBU	BSP 3	BSP3	*B. bubalis* (Domestic water buffalo)	140
9	A0A0K1YXR5	A0A0K1YXR5_BUBBU	BSP 1	BSP1	*B. bubalis* (Domestic water buffalo)	138
10	A0A097GTY0	A0A097GTY0_BUBBU	BSP 1		*B. bubalis* (Domestic water buffalo)	138
11	A0A0M5I4D0	A0A0M5I4D0_BUBBU	BSP 5	BSP5	*B. bubalis* (Domestic water buffalo)	183

From Figure 3, it appears that there are seven variations of the peptide sequence related to the epitope that is possible to be developed as vaccine materials since they are predicted to be antigenic.

In addition to the IEDB program, we also used NetMHCpan-4.1 to evaluate peptide binding affinities between the BSP1 peptides and MHC class I and class II molecules. For this purpose, we searched for murine gene H-2Dq and found one sequence with a strong bind level and three weak bind levels (Table 2). The predicted affinity was classified according to the default NetMHCpan-4.1 program. Strong binding was ≤500 nM, while weak binding was >500 nM, with the rank threshold of strong and weak binding peptides being 0.5% and 2.0%, respectively.

**Figure 4.**
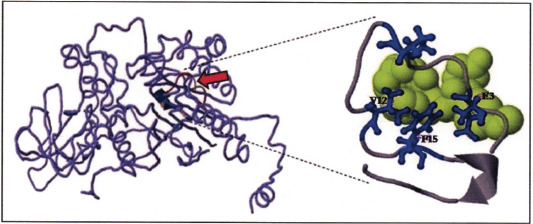
The results of the 3-dimensional theoretical prediction analysis of the peptide binding pattern of BSP1 to its ligand (red arrow), based on the I-TASSER Program. Enlarged exposure image shows the ligand bind site-residues (E3, P7, V12, and F15) of the peptide vaccine LPEDSVPDEERVFPFTYRNRKHF proposed in this study.

We then combined the IEDB data in Figure 3 and NetMHCpan-4.1 (Table 2) and manually determined the vaccine candidate; in this study, i.e., LPEDSVPDEERVFPFTYRNRKHF (sequence of residues number 43–65). This peptide consists of strong and weak binding affinities, with most of them being noticed as antigenic. By using the meta-server approach, COACH, i.e., a combination of COFACTOR, TIM-SITE, and S-SITE programs on I-TASSER, the peptide was predicted in its 3D model along with its ligand-binding site [24]. The result is presented in Figure 4. Based on the PDB hit, the peptide target is close to GP1HB, classified as a DNA-binding protein/DNA. Meanwhile, the first rank for ligand binding site is 3h5bB, related to the crystal structure of wild-type HIV-1 protease, EC 3.4.23.16, known as HIV-1 retropepsin.

## Discussion

The provision of antibodies for developing diagnostic tools for testing male livestock fertility is still limited, and in fact, this is urgently needed. There are several causative factors, including the availability of antigens to make these antibodies. In this study, we tried to develop a peptide-based vaccine from BSP1, which is known as a biomarker of male fertility.

Of these 170 entry identifiers related to BSP1 in the Protein Bank data on ExPASy, UniProtKB, 11 of these numbers are related to ruminants (Table 1). In this study, we used the BSP1 sequence of *B. bubalis* (Domestic water buffalo) as the main target (Fig. 1) and two representations of bovine, i.e., hybrid cattle (*B. indicus* × *B. taurus*) and *B. indicus* (Zebu). As might be expected, the multiple alignment results show that there are several conserved residues among the three (Fig. 2). The expected achievement target in vaccine development in this study is a strong epitope in the conserved part. However, the Emini surface accessibility results combined with NetMHCpanprediction analysis showed that the residue components with potential immunogenic or antigenic properties of buffalo BSP1 were not fully conserved (Fig. 3, Table 2).

**Table 2.** NetMHCpan prediction results of BSP1.

Residue position	MHC	Peptide	Aff. (nM)	Bind level
48	H-2-Dq	VPDEERVFPF	453.42	Strong
39	H-2-Dq	RPAELPEDSV	2,595.69	Weak
74	H-2-Dq	FPWCSLDANY	648.57	Weak
126	H-2-Dq	SPNYDKDRAW	4,302.52	Weak

It is generally based on B cell immunity in vaccine development, although the T cell epitope has also received attention, particularly for CD8+ T cells or MHC-I [27]. This study found that the dominant epitopes are primarily associated with MHC-I T cells or CD8 epitopes (Table 2), compared to B cells and MHC-II T cells (CD4 epitopes). Although the epitope found is mainly related to MHC-I (CD8 T Cells), the sequence of these residues can be appointed as a candidate for peptide vaccine. Several studies have shown that CD8 T cells are essential in inducing immune responses to certain diseases [28–30]. Therefore, we combined the prospective epitopes to make a candidate peptide vaccine consisting of 23 residues, i.e., LPEDSVPDEERVFPFTYRNRKHF.

The results of the three-dimensional structure modeling using I-TASSER showed a similar structure to the HIV-1 retropepsin enzyme (EC 3.4.24.45). When ligand analysis was performed, binding sites were observed at the residues E, P, V, and F, in sequences 3, 7, 12, and 15, respectively, of the peptide vaccine proposed in this study (Fig. 4a), or those of residues numbers 45, 49, 54, and 57 in Figures 1 and 2. Furthermore, When paired with the Emini surface accessibility data in Figure 3, these epitope residues fall into the region above the threshold, which is categorized as immunogenic. The data from I-TASSER was then used to perform molecular docking using MTiOpenScreen [31], and the results obtained (Fig. 4b) strengthened the analysis results of the ligand bonds of the I-TASSER.

Suppose we observed the position of the residues from the epitope of the proposed peptide vaccine. In that case, only F15 (phenylalanine No. 15) or F57 (Fig. 2) is conserved with amino acid sequences from hybrid cattle (*B. indicus* × *B. taurus*) and *B. indicus* (Zebu). This can be understood because *B. bubalis* is different from *B. indicus* and *B. taurus*. Whether in the future, the antibodies produced from the vaccine proposed in this study will produce antibodies specific to the sperm binder only for buffaloes or cross-react with the BSP protein from bulls still needs to be proven *in vivo*.

We sincerely hope that in the near future, we will be able to produce polyclonal antibodies against BSP-1. These antibodies are very important for developing sperm fertility kits or diagnostics for ruminants. The success of making a device to detect whether a male is fertile or infertile will play an important role in the livestock industry [32]. The review of Butler et al. [32] can also consider that male fertility traits can be inherited; hence they can be improved through male selection. Thus, the availability of biomarkers or kits for male fertility indeed plays a central role and is urgently needed.

One of the reasons for the lack of assessment of male quality based on the level of sperm fertility or infertility is the complexity of the male reproductive systems, starting from sperm production and ending with the success of the fertilization process by “selected” sperm in the female animals [33]. On the other hand, Foxcroft et al. [34] acknowledged that the reproductive capacity of males, including their fertility rate, greatly affects the reproductive efficiency of females, namely the impact of pregnancy rates and the number of non-productive days of females. In addition, it is also recognized that detecting bull fertility is extremely difficult, and there is no single test currently available to envisage or diagnose bull sub-fertility or fertility [9,35]. Therefore, the availability of kits to accurately measure bull fertility is very beneficial in managing and strengthening the impact of using superior fertile bulls on genetic advancement and the economic efficiency of artificial insemination programs [32,36]. The results of our study provide opportunities for the development of male fertility detection tools in the future. The results of bioinformatics observations led to the determination of the peptide sequence for the development of the BSP-1 vaccine, which was a peptide with the sequence “LPEDSVPDEERVFPFTYRNRKHF,” a modified part of the BSP-1 sequence in this study.

## Conclusion

The synthetic vaccine from the BSP-1 has been successfully developed with the amino acid sequence LPEDSVPDEERVFPFTYRNRKHF, was buffer-soluble, and the three-dimensional theoretical prediction analysis of the peptide binding pattern of BSP-1 to its ligand, as well as molecular docking, has also been revealed.

## List of abbreviations

BSP-1, Binders of Sperm-1; BSPH1, Binder of sperm protein homolog 1; IEDB, Immune Epitope Database; MALDI-TOF/MS, matrix-assisted laser desorption ionization-time of flight/mass spectrometry; MHC, major histocompatibility complex.

## References

[1] Vernekar VJ, Bandivdekar AH, Raghavan VP, Kamada M, Koide SS (2004). Studies with synthetic peptides of 80 kDa human sperm antigen (80 kDa HSA). AJRI.

[2] Bandivdekar AH (2014). Development of anti fertility vaccine using sperm specific proteins. Indian J Med Res.

[3] Lee BS, Huang JS, Jayathilaka LP, Lee J, Gupta S (2016). Antibody production with synthetic peptides. Methods Mol Biol.

[4] Sadat SM, Aghadadeghi MP, Yousefi M, Khodaei A, Larijani MS, Bahramali G (2021). Bioinformatics analysis of SARS*-*CoV*-*2 to approach an effective vaccine candidate against COVID-19. Mol Biotechnol.

[5] Soria-Guerra RE, Nieto-Gomez R, Govea-Alonso DO, Rosales-Mendoza S (2015). An overview of bioinformatics tools for epitope prediction: implications on vaccine development. J Biomed Inf.

[6] D’Amours O, Bordeleau LJ, Frenette G, Blondin P, Leclerc P, Sullivan R (2012). Binder of sperm 1 and epididymal sperm binding protein 1 are associated with different bull sperm subpopulations. Reproduction.

[7] Rodríguez-Villamil P, Hoyos-Marulanda V, Martins JAM, Oliveira AN, Aguiar LH, Moreno FB (2016). Purification of binder of sperm protein 1 (BSP1) and its effects on bovine *in vitro* embryo development after fertilization with ejaculated and epididymal sperm. Theriogenology.

[8] Rodríguez-Villamil P, Mentz D, Ongaratto FL, Aguiar LH, Rodrigues JL, Bertolini M (2020). Assessment of binder of sperm protein 1 (BSP1) and heparin effects on *in vitro* capacitation and fertilization of bovine ejaculated and epididymal sperm. Zygote.

[9] Özbek M, Hitit M, Kaya A, Jousan FD, Memili E (2021). Sperm functional genome associated with bull fertility. Front Vet Sci.

[10] Westfalewicz B, Dietrich MA, Mostek A, Partyka A, Bielas W, Nizanski W (2017). Analysis of bull (*Bos taurus*) seminal vesicle fluid proteome in relation to seminal plasma proteome. J Dairy Sci.

[11] Feugang JM, Rodriguez-Osorio N, Kaya A, Wang H, Page G, Ostermeier GC (2010). Transcriptome analysis of bull spermatozoa: implications for male fertility. Reprod Biomed Online.

[12] De Oliveira RV, Dogan S, Belser LE, Kaya A, Topper E, Moura A (2013). Molecular morphology and function of bull spermatozoa linked to histones and associated with fertility. Reproduction.

[13] Bromfield JJ (2016). A role for seminal plasma in modulating pregnancy outcomes in domestic species. Reproduction.

[14] Kumar P, Kumar D, Singh I, Yadav PS (2012). Seminal plasma proteome: promising biomarkers for bull fertility. Agric Res.

[15] Viana AGA, Martins AMA, Pontes AH, Fontes W, Castro MS, Ricart CAO (2018). Proteomic landscape of seminal plasma associated with dairy bull fertility. Sci Rep.

[16] Aslam MKM, Sharma VK, Pandey S, Kumaresan A, Srinivasan A, Datta TK (2018). Identification of biomarker candidates for fertility in spermatozoa of crossbred bulls through comparative proteomics. Theriogenology.

[17] Depamede SN (2013). Proteomic analysis of a 14.2 kDa protein isolated from bali cattle (*Bos sondaicus*/*javanicus*) saliva using 1-D SDS-PAGE gel and MALDITOF-TOF mass spectrometer. Italian J Anim Sci.

[18] Madeira F, Park YM, Lee J, Buso N, Gur T, Madhusoodanan N (2019). The EMBL-EBI search and sequence analysis tools APIs in 2019. Nucleic Acids Res.

[19] Vita R, Mahajan S, Overton JA, Dhanda SK, Martini S, Cantrell JR (2018). The Immune Epitope Database (IEDB): 2018 update. Nucleic Acids Res.

[20] Singh H, Raghava GPS (2001). ProPred: prediction of HLA-DR binding sites. Bioinformatics.

[21] Singh H, Raghava GPS (2003). ProPred1: prediction of promiscuous MHC class-I binding sites. Bioinformatics.

[22] Jurtz V, Paul S, Andreatta M, Marcatili P, Peters B, Nielsen M (2017). NetMHCpan-4.0: improved peptide MHC class I interaction predictions integrating eluted ligand and peptide binding affinity data. J Immunol.

[23] Reynisson B, Alvarez B, Paul S, Peters B, Nielsen M (2020). NetMHCpan-4.1 and NetMHCIIpan-4.0: improved predictions of MHC antigen presentation by concurrent motif deconvolution and integration of MS MHC eluted ligand data. Nucleic Acids Res.

[24] Yang J, Zhang Y (2014). I-TASSER server: new development for protein structure and function predictions. Nucleic Acids Res.

[25] Fleri W, Paul S, Dhanda SK, Mahajan S, Xu X, Peters B (2017). The immune epitope database and analysis resource in epitope discovery and synthetic vaccine design. Front Immunol.

[26] Droppa-Almeida D, Franceschi E, Padilha FF (2018). Immune-informatic analysis and design of peptide vaccine from multi-epitopes against *Corynebacterium pseudotuberculosis*. Bioinf Biol Insights.

[27] Hart J, MacHugh ND, Sheldrake T, Nielsen M, Morrison WI (2017). Identification of immediate early gene products of bovine herpes virus 1 (BHV-1) as dominant antigens recognized by CD8 T cells in immune cattle. J Gen Virol.

[28] Rodrigues MM, Boscardin SB, Vasconcelos JR, Hiyane MI, Salay G, Irene Soares S (2003). Importance of CD8 T cell-mediated immune response during intracellular parasitic infections and its implications for the development of effective vaccines. An Acad Bras Cienc.

[29] Oany AR, Sharmin T, Chowdhury AS, Jyoti TP, Hasan A (2015). Highly conserved regions in Ebola virus RNA dependent RNA polymerase may be act as a universal novel peptide vaccine target: a computational approach. In Silico Pharmacol.

[30] Cosma G, Eisenlohr L (2018). CD8 T-cell responses in vaccination: reconsidering targets and function in the context of chronic antigen stimulation. F1000Res.

[31] Labbé CM, Rey J, Lagorce D, Vavrusa M, Becot J, Sperandio O (2015). MTiOpenScreen: a web server for structure-based virtual screening. Nucleic Acids Res.

[32] Butler ML, Bormann JM, Weaber RL, Grieger DM, Rolf MM (2019). Selection for bull fertility: a review. Transl Anim Sci.

[33] Diether N, Dyck MK (2017). Male fertility evaluation using biomarkers in livestock. JSM Biomarkers.

[34] Foxcroft GR, Dyck MK, Ruiz-Sanchez A, Novak S, Dixon WT (2008). Identifying useable semen. Theriogenology.

[35] Castilla JA, Zamora S, Gonzalvo MC, Castillo JDLD, Roldan-Nofuentes JA, Clavero A (2010). Sperm chromatin structure assay and classical semen parameters: systematic review. Reprod Biomed Online.

[36] Nagata MPB, Egashira J, Katafuchi N, Endo K, Ogata K, Yamanaka K (2019). Bovine sperm selection procedure prior to cryopreservation for improvement of post thawed semen quality and fertility. J Anim Sci Biotechnol.

